# *Helicobacter pylori*‐induced YAP1 nuclear translocation promotes gastric carcinogenesis by enhancing IL‐1β expression

**DOI:** 10.1002/cam4.2318

**Published:** 2019-05-30

**Authors:** Yujiao Wu, Li Shen, Xiuming Liang, Shuyan Li, Lin Ma, Lixin Zheng, Tongyu Li, Han Yu, Hillary Chan, Chunyan Chen, Jingya Yu, Jihui Jia

**Affiliations:** ^1^ Department of Microbiology/Key Laboratory for Experimental Teratology of Chinese Ministry of Education, School of Medicine Shandong University Jinan P. R. China; ^2^ Department of Hematology Qilu Hospital, Shandong University Jinan Shandong P. R. China; ^3^ The Faculty of Medicine The University of Toronto Toronto Canada

**Keywords:** gastric cancer, *Helicobacter pylori*, IL‐1β, TEAD, YAP1

## Abstract

Gastric cancer (GC) is one of the most common and malignant pathologies, and a significant portion of GC incidences develops from *Helicobacter pylori (Hp)*‐induced chronic gastritis. Although the exact mechanisms of GC are complex and poorly understood, gastric carcinogenesis is a good model to investigate how inflammation and infection collaboratively promote tumorigenesis. Yes‐associated protein 1 (YAP1) is the key effector of the Hippo pathway, which is silenced in most human cancers. Herein, we verified the tumor‐promoting effect of YAP1 in vitro, in vivo, and in human specimens. We revealed that YAP1 displays nuclear translocation and works with TEAD to activate transcription of the crucial inflammatory cytokine IL‐1β in gastric cells infected with *Hp*. As IL‐1ß accounts for inflammation‐associated tumorigenesis, this process can lead to gastric carcinogenesis. Thus, in addition to activating proliferation genes, YAP1 also plays a major role in inflammation amplification by activating inflammatory cytokine genes. Excitingly, our research demonstrates that transfection of mutant plasmid YAP‐5SA/S94A or addition of the drug verteporfin, both of which are thought to disrupt the YAP1‐TEAD interaction, can arrest the carcinogenesis process. These findings can provide new approaches to GC treatment.

## 1 INTRODUCTION

With one of the five highest morbidities among all cancer types, gastric cancer (GC) is also one of the top five causes of cancer death and led to 951 600 new cases and approximately 723 100 deaths in 2012.^1^ *Helicobacter pylori* (*Hp*) infection plays a triggering role in the process of inflammation‐associated gastric carcinogenesis.^2^ *Hp* is such a successful pathogenic microorganism that approximately 50% of all human stomachs have been colonized.^3^ Recent studies have shown that *Hp* chronic infection accounts for approximately 90% of new noncardia GC cases worldwide, ranking it the most dangerous risk factor for GC. Interactions between *Hp* and its hosts are rather complicated. *Hp* uses its bacterial type IV secretion system (T4SS) to inject the toxin CagA into its host's stomach epithelial cells, triggering a vast range of downstream signaling cascades and ultimately accelerating gastric carcinogenesis.^4^ Other toxins, such as VacA and CagL, as well as *Hp* components, such as lipopolysaccharides (LPS), also contribute to this malignant transformation.

Yes‐associated protein 1 (YAP1) is the key terminal effector of the Hippo pathway, an old, conserved pathway that was first found to regulate organ size and cell fate that responds to density signals outside the membrane.^5^, ^6^ YAP1 is normally inactive in the cytoplasm due to the phosphorylation of serine 127 by the upstream kinase LATS.^7^ When extracellular signals silence the Hippo cascade, as the terminal effector, YAP1 is translocated into the nucleus and becomes a transcription co‐activator, cooperating with transcription factors, such as TEAD family members, to initiate the transcription of multiple oncogenes.^8^, ^9^ YAP1 overexpression has been reported in various human cancers, including breast, lung, colorectal, ovarian, pancreatic, gastric, and liver cancer.^9^ In addition, the promotional role of YAP1 nuclear translocation has been indicated in different cancers, including hepatocellular carcinoma, non‐small‐cell lung cancer, and breast cancer.^10^, ^11^, ^12^ In GC, elevated YAP1 and its nuclear accumulation are associated with poor prognosis.^13^, ^14^, ^15^ YAP1 has been shown to promote proliferation and metastasis as well as to induce apoptosis.^16^ However, the molecular mechanisms underlying how YAP functions in gastric carcinogenesis require further elucidation.

IL‐1β is a multifunctional proinflammatory cytokine that has profound inflammatory and immune effects,^17^ and it plays a crucial role in the initiation and development of a wide range of inflammation‐associated cancers,^18^, ^19^, ^20^ especially GC.^17^, ^18^ IL‐1β is widely reported to promote gastric carcinogenesis and is associated with poor prognosis.^21^, ^22^, ^23^, ^24^ IL‐1β polymorphisms (IL‐1b‐511T and IL‐1b‐31C) can promote GC by boosting IL‐1β production and increasing circulating cytokine levels,^17^, ^18^ and *Hp* infection can induce IL‐1β expression.

This study aimed to prove that YAP1 plays a tumor‐promoting role in *Hp*‐induced gastric carcinogenesis via activating the key cancer‐related inflammatory cytokine IL‐1β and thereby provide a new drug target for GC treatment.

## 2 MATERIALS AND METHODS

### 2.1 Cell lines and culture

The immortal gastric epithelium cell line GES‐1 and the GC cell lines AGS and BGC‐823 were used in this study. Cells were cultured in RPMI‐1640 medium (Gibco) with 10% fetal bovine serum. All cells were incubated at 37°C in a humidified 5% CO2 atmosphere.

### 2.2 Clinical samples and datasets

Twenty‐eight human AG samples and thirteen GC samples were obtained from surgery patients. The specimens were collected immediately after surgery and stored in formalin. The diagnosis of GC for all patients was confirmed by histological examination. Eighty‐two AG samples were obtained from a gastro‐endoscope room. All patients had pathology reports from the hospital.

The TCGA Research Network is available at http://cancergenome.nih.gov/. GTEx dataset analysis is available at https://www.gtexportal.org/home/. Kaplan‐Meier plotter is available at http://kmplot.com/.^25^

### 2.3 *Helicobacter pylori (Hp)* culture

*Hp* strains were cultured in Brucella broth with 5% fetal bovine serum under microaerobic conditions (5% O_2_, 10% CO_2_, and 85% N_2_) at 37°C. Bacteria were harvested and centrifuged and then added to gastric cell lines at varying multiplicity of infection (MOI) ratios.

### 2.4 Animal experiments

Seven (four weeks old) male nude mice were purchased from QING ZI LAN Animal Company (Nanjing, China). One week after their arrival, seven five‐week‐old nude mice were used for xenograft experiments with both control and shYAP1 BGC‐823 cells. In total, 4 × 10^5 ^BGC‐823 cells were subcutaneously injected for each treatment. Tumors could be observed beginning on day 8, and relative parameters were recorded to construct the tumor growth curve. All the mice were sacrificed on day 22, and their tumors were collected for both mRNA extraction and IHC.

### 2.5 siRNA, transfections, and lentivirus infection

YAP1, IL‐1β, and negative control (nc) siRNAs were purchased from the RiboBio company (the si‐h‐YAP1 and si‐h‐IL‐1β kits each included three target sequences). We transfected AGS, BGC‐823, and GES‐1 cells with 20 nmol/L siRNA in antibiotic‐free Opti‐MEM (Gibco) for 72 hours using Lipofectamine 2000 (Invitrogen) according to the manufacturer's instructions. The shYAP1 lentivirus was produced by the GENECHEM company using the siYAP1 sequences 5’‐GGUGAUACUAUCAACCAAAdTdT‐3’ and 3’‐dTdT CCACUAUGAUAGUUGGUUU‐5’.

### 2.6 Plasmids

pcDNA4/HisMaxB‐YAP1‐S127A (Addgene plasmid #18988), pcDNA4/HisMaxB‐YAP1 (Addgene plasmid #18978), and pCMV‐flag YAP2‐5SA (Addgene plasmid #27371) were obtained from Addgene. The pGL3 Basic luciferase reporter vector was purchased from Promega.

### 2.7 Colony formation assay

We transfected AGS, BGC‐823, and GES‐1 cells with siRNA (siYAP1 and siIL‐1β) or plasmids. Seventy‐two hours after siRNA transfection or forty‐eight hours after plasmid transfection, the cells were seeded in 6‐well plates. For AGS cells, seven days were needed for the colonies to grow, while 14 days were needed for the BGC‐823 and GES‐1 cells. After 7‐14 days of incubation at 37°C in a humidified 5% CO2 atmosphere, the colonies were fixed and stained with methanol and Giemsa buffer, respectively, and the colony numbers were counted.

### 2.8 Western blotting

Cell pellets from the different treatment groups were lysed with 1 × Laemmli sample lysis buffer (62.5 mmol/L Tris‐HCl (pH 6.8), 10% glycerol, 2% SDS, 0.002% bromophenol blue, and 100 mmol/L DTT), and a spectrophotometer was used to determine the protein concentrations. We used the Bio‐Rad system to run electrophoresis and transfer proteins to PVDF membranes. After 1.5 hours of blocking (blocking buffer: 5% milk diluted in 1 × TBST buffer), primary antibodies were added, and the membranes were incubated at 4°C overnight with gentle shaking. The secondary antibodies were horseradish peroxidase‐labeled, and Millipore ECL reagents were used to detect the proteins on the blots.

### 2.9 Antibodies

YAP (D8H1X) XP® rabbit mAb (#14074) and phospho‐YAP (Ser127) (D9W2I) rabbit mAb (#13008) were purchased from Cell Signaling Technology (CST), and the IL‐1β antibody (16806‐1‐AP) was purchased from Proteintech. The anti‐CagA antibody was purchased from Abcam, and the β‐Actin antibody (C4) (sc‐47778) was purchased from Santa Cruz Biotechnology.

### 2.10 Immunofluorescence (IF) staining

Cells were initially seeded on coverslips. After treatment and incubation in the cell culture incubator, the cells were fixed in 4% paraformaldehyde and incubated with primary antibodies at 4°C overnight. Anti‐rabbit IgG (H + L) and the F(ab’)2 fragment (CST) were used as secondary antibodies, and DAPI (4‐6‐diamidino‐2‐phenylindole dihydrochloride) was used to detect nuclei.

### 2.11 Edu assay

The Cell‐LightTM EdU Apollo®488 In Vitro Imaging Kit was used to detect cell proliferation according to the manufacturer's instructions (Guangzhou RiboBio).

### 2.12 Immunohistochemistry (IHC) staining

Tissues from patients and mice were embedded in paraffin. After deparaffination, dehydration, epitope retrieval, and H2O2 treatment, the sections were blocked in 5% normal goat serum for 30 min and incubated with primary antibodies at 4°C overnight. After incubation with the secondary antibody, the DAB Staining Kit (Vector Laboratories) was used for staining.

### 2.13 RNA extraction and quantitative RT‐PCR

Total RNA was extracted from harvested cells using TRIzol reagent according to the manufacturer's instructions, and the RevertAid First Strand DNA Synthesis Kit (Fermentas) was used to reverse transcribe the total RNA into cDNA. The sequences of primers used in the qPCR assays were as follows: β‐actin: 5’‐AGTTGCGTTACACCCTTTCTTG‐3’ and 5’‐CACCTTCACCGTTCCAGTTTT‐3’; and YAP1‐168:5’‐CATGGCAGAAAGACTGAAAAATAAC‐3’ and 5’‐GAGGATAAAATCCACCTGAGCAC‐3’.

### 2.14 Dual luciferase assay

Human IL‐1β promoter fragments were cloned from human genomic DNA by PCR. Primers containing the KpnI and HindIII restriction sites were designed in the fragment sequences to construct luciferase reporter plasmids. The primers were as follows: IL‐1‐1 (motif A) F: 5’‐GGTACCCCCCAGCCAAGAAAGGTCA‐3’, R: 5’‐AAGCTTTGGAAGGGCAAGGAGTAGC‐3’; and IL‐1‐2 (motif B) F: 5’‐5’‐GGTACCACAACAGGCTGCTCTGGGATT‐3’, R: 5’‐AAGCTTGGAGCAGAGGCTTTGACACTAA‐3’. The fragments were cloned into the pGL3 Basic luciferase reporter vector (Promega). The Luciferase Assay System (Promega) was used to detect firefly and Renilla luminescence, and the Renilla signal was used for normalization.

### 2.15 Chromatin immunoprecipitation (ChIP) assay

A Chromatin Immunoprecipitation (ChIP) Assay Kit (Millipore) was used to perform the ChIP assay. Sonicated chromatin fragments were incubated overnight at 4°C with the anti‐YAP1 antibody, and bound DNA precipitants were analyzed using PCR. The primers used for PCR detection were as follows: IL‐1‐1 (motif A) F: 5’‐GGTACCCCCCAGCCAAGAAAGGTCA‐3’ and R: 5’‐AAGCTTTGGAAGGGCAAGGAGTAGC‐3’; and IL‐1‐2 (motif B) F: 5’‐GGTACCACAACAGGCTGCTCTGGGATT‐3’ and R: 5’‐AAGCTTGGAGCAGAGGCTTTGACACTAA‐3’.

### 2.16 Statistical analysis

Experimental quantitative data were obtained in biological replicates and shown as means (±SD). Student's *t* tests, χ^2^ tests, and log‐rank test were used to analyze the data. *P* < 0.05 was considered statistically significant.

## 3 RESULTS

### 3.1 YAP1 is upregulated in human gastric specimens and gastric cell lines

**Figure 1:**
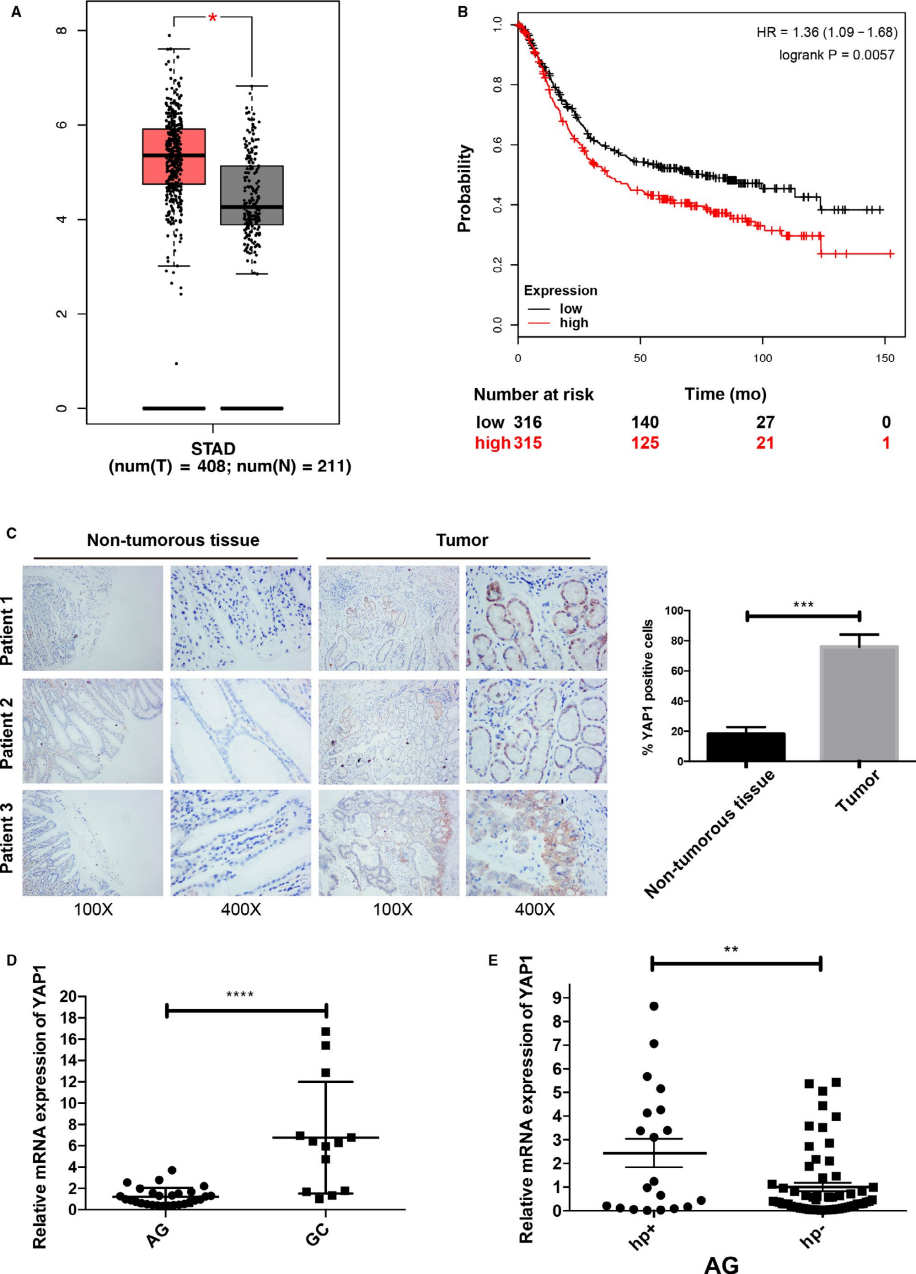
YAP1 expression in gastric cancer and its association with *Helicobacter pylori* infection. (A) TCGA (https://cancergenome.nih.gov) and GTEx (https://www.gtexportal.org/home/) data analysis of YAP1 mRNA levels in normal gastric mucosa tissues and stomach adenocarcinoma (STAD) tissues (Stomach Adenocarcinoma (TCGA, Provisional) January 2017). Normal gastric mucosa has lower YAP1 expression. (B) http://kmplot.com/ data analysis showed higher YAP1 expression is a predictor for shorter overall survival in GC patients. The median cutoff was used to categorize patients into low and high YAP1 expression groups. Mantel‐Cox test was used in the survival analysis. Hazard ratio (HR) was presented in each panel, respectively. (C) Immunohistochemical staining was performed on nontumorous gastric samples and gastric tumor samples. Quantification of the data shows that YAP1 is highly expressed in gastric tumor tissues (data are presented as the mean ± SD, ***=*P* < 0.001). (D) Relative YAP1 mRNA levels were detected by qPCR in atrophic gastritis (AG, n = 28) and gastric cancer (GC, n = 13) patient samples (data are presented as the mean ± SD, ****=*P* < 0.0001, unpaired *t* test). (E) Atrophic gastritis (AG) patient samples were divided into *Hp*+ (n = 20) and *Hp*‐ (n = 62) groups based on *Helicobacter pylori (Hp)* infection. YAP1 mRNA levels were detected by qPCR (data are presented as the mean ± SD, *=*P* < 0.05, **=*P* < 0.01, unpaired *t* test)

To clarify the association between YAP1 and GC, we first collected data from the TCGA (The Cancer Genome Atlas) and GTEx (Genotype‐Tissue Expression) database and compared the YAP1 mRNA expression levels among normal gastric tissues (N = 211) and cancerous gastric tissues (T = 408)^26^ (Figure 1A). YAP1 was expressed at significantly higher levels in stomach adenocarcinoma (STAD) than in normal gastric mucosa. Kaplan‐Meier analysis revealed that high YAP1 expression was significantly associated with shorter patient overall survival in GC (Figure 1B). We next stained YAP1 in paired nontumorous and cancerous gastric tissues using immunohistochemistry (IHC) (Figure 1C), revealing that both the positivity and intensity of the YAP1 signals were enhanced in GC. We detected YAP1 expression levels in 28 human atrophic gastritis (AG) samples and 13 GC samples by quantitative real‐time PCR (qPCR), revealing obviously higher YAP1 expression in GC tissues compared to that in AG tissues (Figure 1D). YAP1 expression in GC reached a new level, which emphasized the role of YAP1 in AG‐GC malignant transformations. We next divided 82 AG tissue samples into *Hp*‐positive and *Hp*‐negative groups. We performed qPCR and IHC staining and found high YAP1 expression levels in *Hp*‐positive patients, which implied the role of YAP1 in *Hp*‐induced gastric malignancy progression (Figure 1E and Figure S1A). Higher levels of YAP1 expression were detected in the GC cell lines AGS and BGC‐823 compared with that in the gastric epithelial cell line GES‐1 (Figure S2A). These results illustrated that YAP1 is overexpressed in human gastric tumor tissues and might be positively correlated with *Hp*‐induced GC progression.

### 3.2 *Helicobacter pylori* promotes YAP expression and nuclear translocation

**Figure 2:**
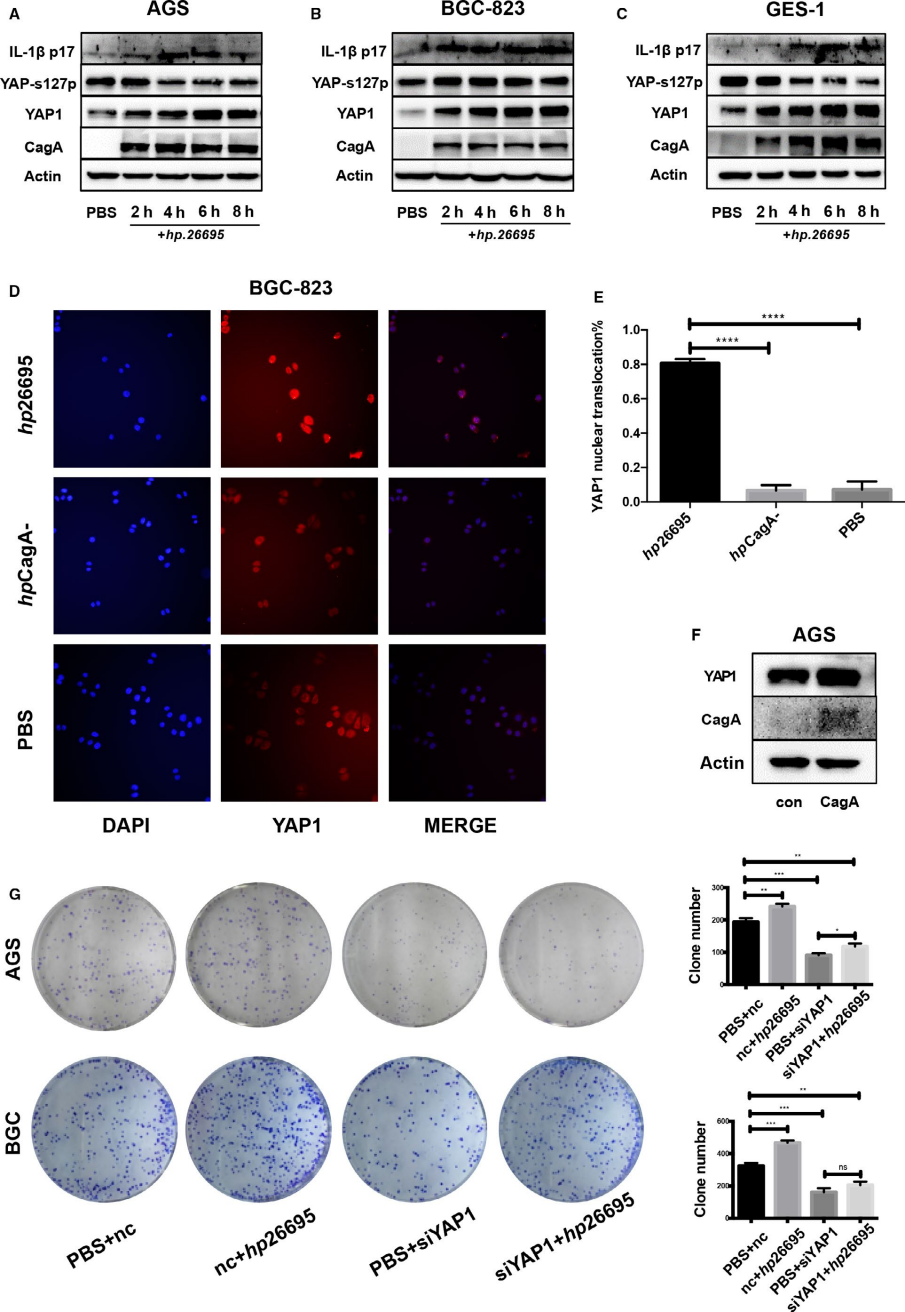
*Helicobacter pylori* infection promotes proliferation and inflammation and induces YAP1 overexpression and nuclear translocation. (A, B, C) PBS and *hp26695* were added to AGS, BGC‐823, and GES‐1 cells, respectively (MOI = 100:1). The cells were harvested at 2, 4, 6, and 8 h, and Western blot analysis was performed. YAP1 and IL‐1β expression increased, while phosphorylation at the S127A position on YAP1 was decreased in a time‐dependent manner. (D, E) Immunofluorescence staining of BGC‐823 cells infected with CagA‐positive and CagA‐negative *Hp* strains, respectively (****=*P* < 0.0001, unpaired *t* test). (F) YAP1 was upregulated in AGS cells overexpressing CagA. (G) Colony formation of AGS and BGC cells. PBS or *hp26695 *was added to cells 72 h after knocking down YAP1. Quantification is shown at the bottom (data are presented as the mean ± SD of three independent experiments, *=*P* < 0.05, **=*P* < 0.01, ***=*P* < 0.001, unpaired *t* test)

In our studies, the fact that *Hp* might be associated with YAP1 overexpression aroused our attention. Additionally, we found an interesting phenomenon that *Hp* infection can promote YAP1 expression and nuclear translocation. We added the *Hp* standard strain *hp*26695 to the gastric epithelial cell lines AGS and BGC‐823 and the human gastric epithelial cell line GES‐1 and harvested these cells at 0, 2, 4, 6, and 8 hours. We then detected the protein expression of YAP1 and YAP1 serine 127 phosphorylation (YAP1S127) by Western blot (Figure 2A‐C). YAP1S127 is a phosphorylated form of YAP1 that cannot be transferred into the nucleus. With *Hp*26695 infection, YAP1 protein expression in AGS, BGC‐823, and GES‐1 cells was upregulated in a time‐dependent manner, while YAP1S127 protein expression showed the opposite trend, indicating a potential increase in YAP1 nuclear translocation. To investigate whether the *Hp*26695 and CagA stimuli could trigger YAP1 nuclear translocation, we conducted immunofluorescence (IF) staining after infecting AGS and BGC‐823 cells with the CagA+ and CagA− *Hp* strains. *Hp* promoted YAP1 nuclear translocation only when CagA was present, as the CagA− *Hp* infection did not show similar effects (Figure 2D,E). We then transfected AGS cells with the CagA plasmid, and the results were consistent (Figure 2F). To investigate the role of YAP1 in *Hp*‐infected GC cells, we knocked down YAP1 using YAP1 small interfering RNA (siRNA). Among three parallel YAP1 siRNAs, siYAP1.3 (si3) had the highest knockdown efficiency (Figure S2B) and was thus used to perform all knockdown experiments in this study. Seventy‐two hours after the YAP1 siRNA was transfected, AGS and BGC‐823 cells were infected with *hp*26695 for 6 hours, and the colony formation assay was then performed (Figure 2G). In both cell lines, *Hp*26695 infection significantly increased the colony formation ability, and this effect was inhibited by YAP1 knockdown. These results proved that *Hp* promotes GC cell proliferation partially by regulating YAP1. *Hp* functions not only by upregulating YAP1 expression but also by promoting YAP1 nuclear translocation. *Hp* enhanced YAP1 expression and nuclear translocation by injecting its toxin protein CagA into gastric epithelial cells.

### 3.3 YAP contributes to GC cell proliferation in vitro

**Figure 3:**
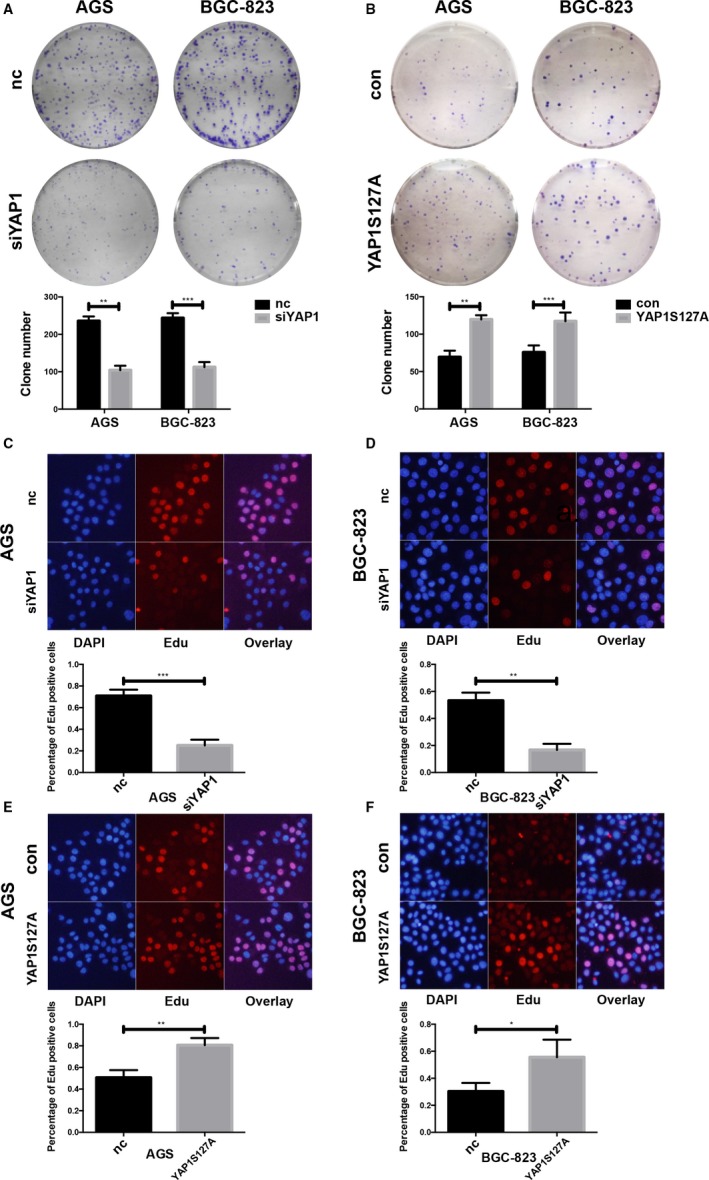
YAP1 promotes gastric cancer cell proliferation in vitro. (A) The clonogenic abilities of AGS and BGC cells were decreased upon YAP1 knockdown (data are presented as the mean ± SD of three independent experiments, **=*P* < 0.01, ***=*P* < 0.001, unpaired *t* test). (B) The clonogenic abilities of AGS and BGC cells were increased upon overexpression of the nuclear located form of YAP1 (YAP1S127A) (data are presented as the mean ± SD of three independent experiments, **=*P* < 0.01, ***=*P* < 0.001, unpaired *t* test). (C, D) Edu assay showing the reductive proliferative abilities of AGS and BGC cells upon YAP1 knockdown (**=*P* < 0.01, ***=*P* < 0.001). (E, F) Edu assay showing the enhanced proliferative abilities of AGS and BGC cells upon YAP1S127A transfection (*=*P* < 0.05, **=*P *< 0.01)

To further investigate the role of YAP1 in GC cell proliferation, we induced YAP1 siRNA in the AGS and BGC‐823 cell lines and performed colony formation assays. Both AGS and BGC‐823 cells had concordant results; cells of the siYAP1 group formed much fewer colonies than those of the nc group (Figure 3A). The YAPS127 plasmid provides sustained overexpression of the serine 127 mutant form of YAP1. This YAP1 mutant cannot be phosphorylated at its serine 127 position and thus can continuously translocate into the nucleus. We transfected AGS and BGC‐823 cells with the YAPS127 mutant plasmid and performed the colony formation assay (Figure 3B). Both AGS and BGC‐823 control group cells formed fewer colonies than the YAPS127 group, indicating that activated YAP1 plays a key role in GC cell proliferation. The efficiency of the plasmid was confirmed by Western blotting (Figure S2C, D). We also collected YAP1 knockdown and YAP1 activated cells to test their proliferation capacities using the Edu assay. In accordance with the colony formation assays, the Edu assays also provided evidence of the promotional effect of YAP1 on proliferation in vitro (Figure 3C‐F), suggesting that YAP1 can transform non‐tumorigenic cells in vitro. These results collectively prove that YAP1 can contribute to GC cell proliferation and might promote the malignant transformation of nontumorigenic gastric epithelial cells.

### 3.4 YAP1 can induce gastric tumor growth in vivo

**Figure 4:**
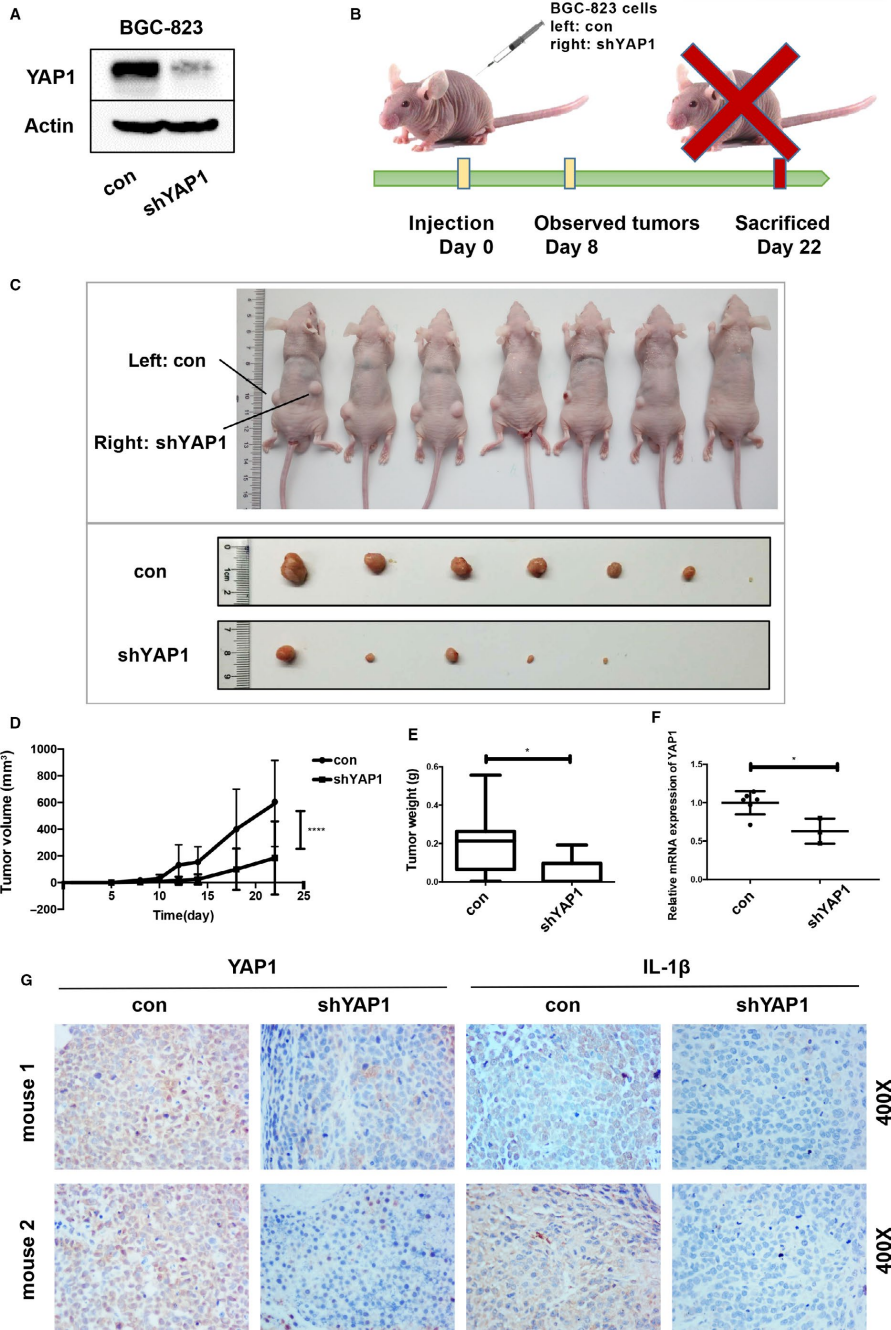
YAP1 promotes gastric tumorigenesis in vivo. (A) Stable YAP1 knockdown was performed in the BGC‐823 cell line using a lentivirus system. The knockdown efficiency was confirmed by Western blot. (B, C) Tumors in nude mice generated by BGC‐823 cells transfected with a negative control (nc, left side) and lenti‐shYAP1(shYAP1, right side). (D) Tumor growth curve showing a lower growth rate after YAP1 knockdown. (E) Tumor weight. Tumors were collected after the mice were sacrificed. Tumors from the negative control and shYAP1 groups were weighed (data are presented as the mean ± SD, *=*P* < 0.05, unpaired *t* test). (F) YAP1 mRNA levels in tumors were detected by qPCR (data are presented as the mean ± SD, *=*P* < 0.05, unpaired *t*‐test). (G) IHC results showing decreased YAP1 and IL‐1β expression levels in the shYAP1 group compared with those in the nc group

To confirm the oncogenic role of YAP1 in gastric epithelial cells in vivo, we constructed a lentivirus‐infected BGC‐823 cell line stably expressing shYAP1. The YAP1 knockdown efficiency was verified at the protein level (Figure 4A). Both negative control (con) and shYAP1 BGC‐823 cells were used for xenograft experiments with subcutaneous infection. Tumors could be observed beginning on day 8, and relative parameters were recorded to construct the tumor growth curve (Figure 4B,D). All of the mice were sacrificed on day 22, and their tumors were excised (Figure 4C). According to the photographs shown in Figure 4C, the shYAP1 group formed smaller and fewer (con/n = 7, shYAP1/n = 5) tumors than the negative control group. Tumors from the shYAP1 group also had reduced volumes and weights compared to those of the control group, which was in accordance with the result in Figure 4C (Figure 4D,E). The tumors were collected for both RNA extraction and paraffin sectioning. YAP1 mRNA expression was significantly downregulated in the shYAP1 group (Figure 4F), and IHC analysis indicated a similar trend (Figure 4G). Moreover, decreased expression of IL‐1β, a crucial inflammatory cytokine, was also proven by IHC analysis (Figure 4G). Hematoxylin and eosin (HE) stain showed that more lymphocytes were observed in the negative control group than shYAP1 group, indicating reduced inflammation in the xenografts with YAP1 knockdown (Figure S1B). The collected data confirmed YAP1’s role in the initiation and growth of tumors generated from GC cells in vivo.

### 3.5 YAP1 regulates IL‐1β transcription directly in cooperation with TEAD

**Figure 5:**
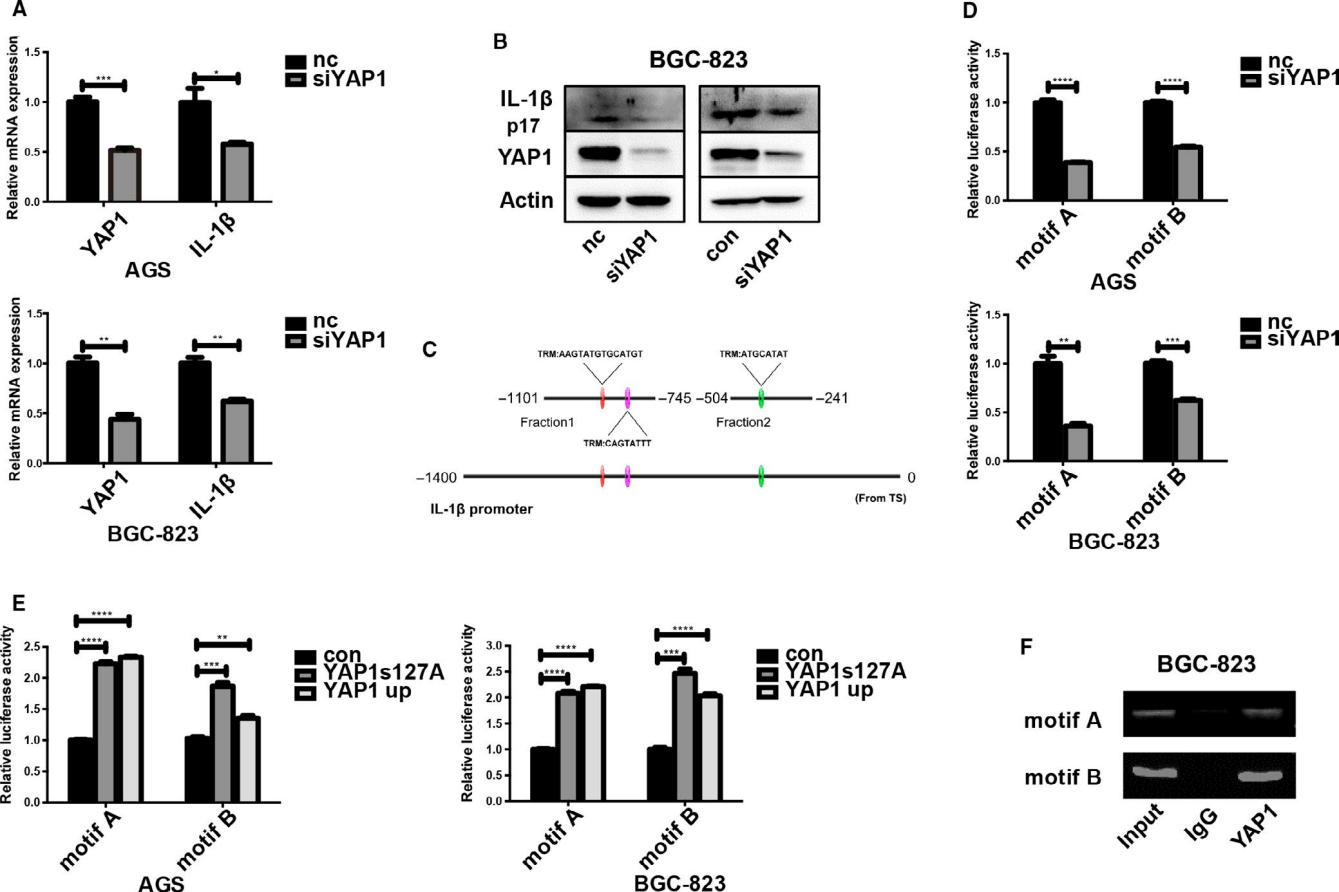
YAP1 induces IL‐1β expression by binding the IL‐1β promoter region. (A) Loss of YAP1 reduced IL‐1β mRNA expression in AGS and BGC‐823 cells (data are presented as the mean ± SD of three independent biological experiments, *=*P* < 0.05, ***=*P* < 0.001, unpaired *t* test). (B) IL‐1β was downregulated when YAP1 was knocked down in YAP1 siRNA‐transfected and stable shYAP1 BGC‐823 cells. (C) TEAD binding sites on the IL‐1β promoter region. Three TEAD binding sites were divided into two motifs, which were inserted into a backbone plasmid for the construction of a dual luciferase assay vector. (D) Decreased luciferase activity (motif A and motif B) was observed upon YAP1 knockdown in both AGS and BGC‐823 cells (data are presented as the mean ± SD, **=*P* < 0.01, ***=*P* < 0.001, ****=*P* < 0.0001). (E) Increased luciferase activity (motif A and motif B) was observed in AGS and BGC‐823 cells transfected with the YAP1S127A and YAP1up plasmids (data are presented as the mean ± SD, **=*P* < 0.01, ***=*P* < 0.001, ****=*P* < 0.0001). (F) ChIP analysis confirming that YAP1 can bind two regions of the IL‐1β promoter in BGC‐823 cells

IHC staining of mouse tumor tissues demonstrated decreased IL‐1β expression in the shYAP1 group (Figure 4G). A time‐dependent increase in IL‐1β expression was also observed in the *Hp* infection experiment, following the trend of YAP1 (Figure 2A‐C). We knocked down YAP1 expression in the GC cell lines AGS and BGC‐823 using YAP1 siRNA and detected IL‐1β mRNA expression by qPCR, observing that IL‐1β mRNA expression was decreased by approximately 50% after YAP1 knockdown (Figure 5A). Similar YAP1 knockdown results were obtained in BGC‐823 cells at the protein level using both siYAP1 and shYAP1 (Figure 5B). Because numerous published studies have reported that YAP1 can activate downstream genes as a transcriptional co‐activator by interacting with TEAD family transcription factors, we proposed that YAP1 regulates IL‐1β transcription directly by binding to IL‐1β’s promoter region in cooperation with TEAD. According to T Mizuno's publication in Oncogene in 2012,^8^ we searched the classic putative TEAD recognition motif on IL‐1β’s promoter region and found three TEAD DNA‐binding sites within 1000‐bp upstream of the TSS of IL‐1β (Figure 5C). Since binding sites 1 and 2 were close to each other, we regarded the two sites together as motif A. Motif B was located between Motif A and the TSS. Motifs A and B were constructed into two plasmids for dual luciferase assays, which were conducted to verify that YAP1 functions to activate IL‐1β gene expression. The luciferase signals in the siYAP1‐transfected group were weaker than those in the nc group in both AGS and BGC‐823 cells (Figure 5D), and the luciferase activity was stronger in both cell lines transfected with the high YAP1 expression plasmid YAP1up and the serine 127 mutant plasmid YAP1S127 compared with that in the control group (Figure 5E). These findings were then validated with a chromatin immunoprecipitation (ChIP) assay. DNA immunoprecipitated by a YAP1 antibody contained the proximal promoter with two putative TEAD binding motifs on the IL‐1β gene, which was confirmed by PCR (Figure 5F). These results indicated that YAP1 can bind the IL‐1β promoter as a transcription co‐factor and thus regulate IL‐1β production.

**Figure 6:**
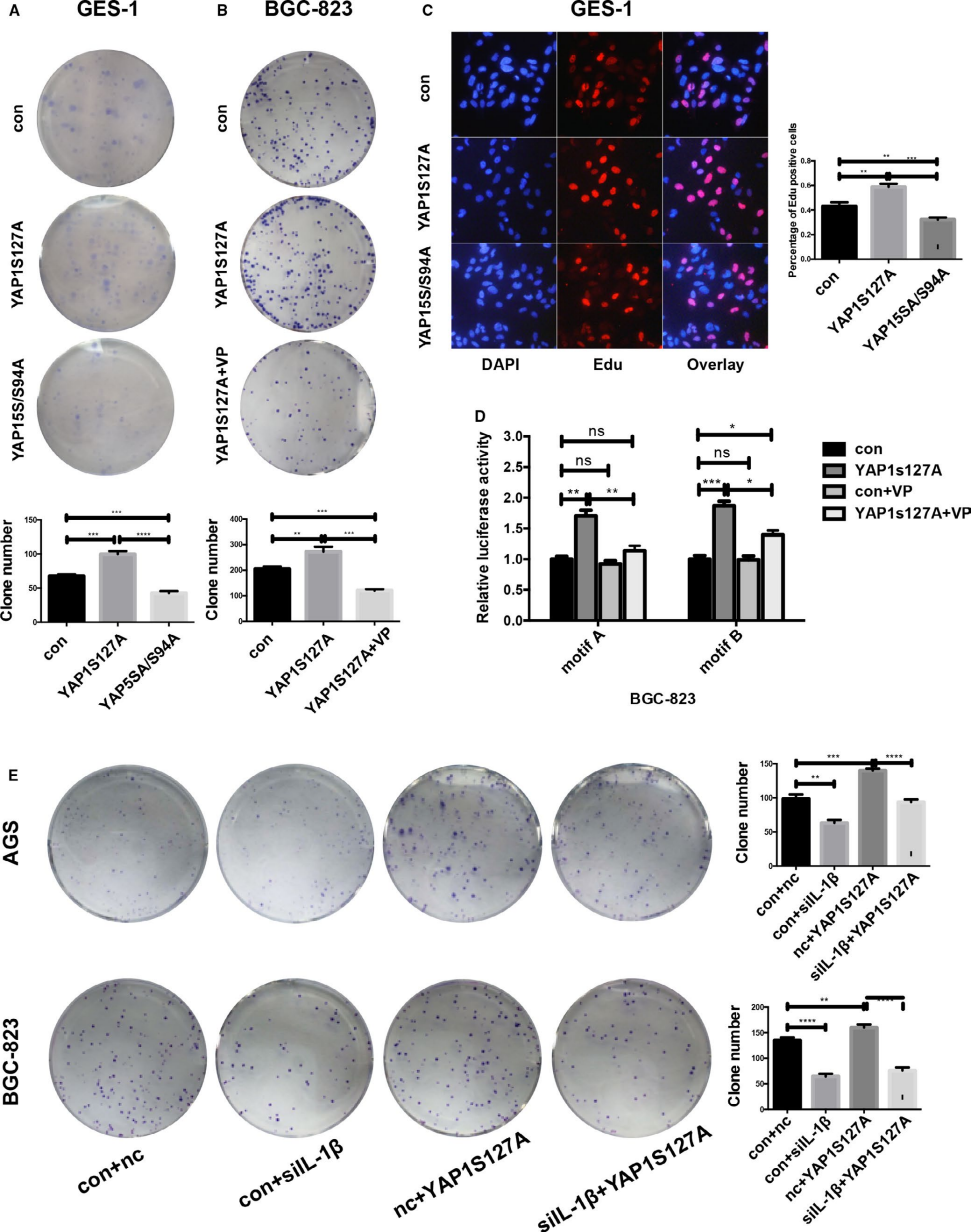
YAP1 functions on IL‐1β promoter via interaction with the transcriptional factor TEAD. (A) GES‐1 cells transfected with the YAP5SA/S94A plasmid formed fewer colonies than the YAP1S127A‐transfected and control groups (data are presented as the mean ± SD, ***=*P* < 0.001, unpaired *t* test). (B) Verteporfin (VP, 10 μmol/L) reversed the promotional proliferation effect of YAP1 on BGC‐823 cells (data are presented as the mean ± SD, **=*P* < 0.01, ***=*P* < 0.001, unpaired t test). (C) Edu assay showing that the YAP5SA/S94A plasmid can compromise the promotional proliferation effect of YAP1 on GES‐1 cells. (D) Dual luciferase assay showing that VP (10 μmol/L) disrupted the regulation of YAP1 on the IL‐1β promoter region (data are presented as the mean ± SD, *=*P* < 0.05, **=*P* < 0.01, ***=*P* < 0.001). (E) Loss of IL‐1β partially compromises the pro‐proliferative effect of YAP1. Complementary experiments were performed in AGS and BGC‐823 cells by transfecting the YAP1S127A plasmid into IL‐1β knockdown cells. IL‐1β knockdown compromised the positive effect of YAP1s127A on colony formation (data are presented as the mean ± SD, **=*P* < 0.01, ***=*P* < 0.001, ****=*P* < 0.0001)

YAP1‐5SA and YAP1‐S94A are mutant forms of YAP1. YAP1‐5SA expresses constitutively active YAP1‐5SA, which is resistant to phosphorylation at multiple sites by Mst2 and Lats2. YAP1‐S94A can disrupt the YAP1‐TEAD interaction but does not impair its general transcriptional activity. Here, we introduced the YAP1‐5SA/S94A mutant plasmid, which is a combination of YAP1‐5SA and YAP1‐S94A, to explore the role of TEAD in IL‐1β activation 27 (TEAD mediates YAP1‐dependent gene induction and growth control). We transfected GES‐1 cells with the YAP1S127A and YAP‐5SA/S94A mutant plasmids. Colony formation and Edu assays were performed after 72 hours, revealing that YAP‐5SA/S94A could reverse the positive effect of YAP1S127A on proliferation (Figure 6A,C). These results showed that when the YAP1‐TEAD interaction was disrupted, GES‐1 cells had a decreased proliferation ability even if the active form of YAP1 was continuously overexpressed, which indicated that TEAD plays a key role in assisting YAP1’s activity on the IL‐1β promoter.

Verteporfin (VP), a drug that was first reported to treat neovascular age‐related macular degeneration that is capable of disrupting YAP1‐TEAD binding,^28^, ^29^, ^30^ was utilized to reveal the function of YAP1‐TEAD in initiating IL‐1β transcriptional expression. VP was dissolved in DMSO and added at 10 μmol/L 24 hours after BGC‐823 cells were transfected with the YAP1S127A plasmid. The colony formation assay showed that VP compromised the ability of YAP1S127A to promote proliferation (Figure 6B). Similarly, cells treated with VP had a weaker luciferase signal than those transfected with the YAP1S127A plasmid (Figure 6D), suggesting that VP slows GC cell proliferation by disrupting the YAP1‐TEAD interaction.

These findings confirmed YAP1’s transcription co‐activator role in the regulation of IL‐1β and elucidated the mechanism of this process. Transfection of mutant plasmids or addition of the drug VP, which disrupts the YAP1‐TEAD interaction, can arrest the carcinogenesis process. Our findings might provide a new drug target for GC treatment.

### 3.6 Deleting IL‐1β partially neutralized YAP's ability to promote proliferation

**Figure 7:**
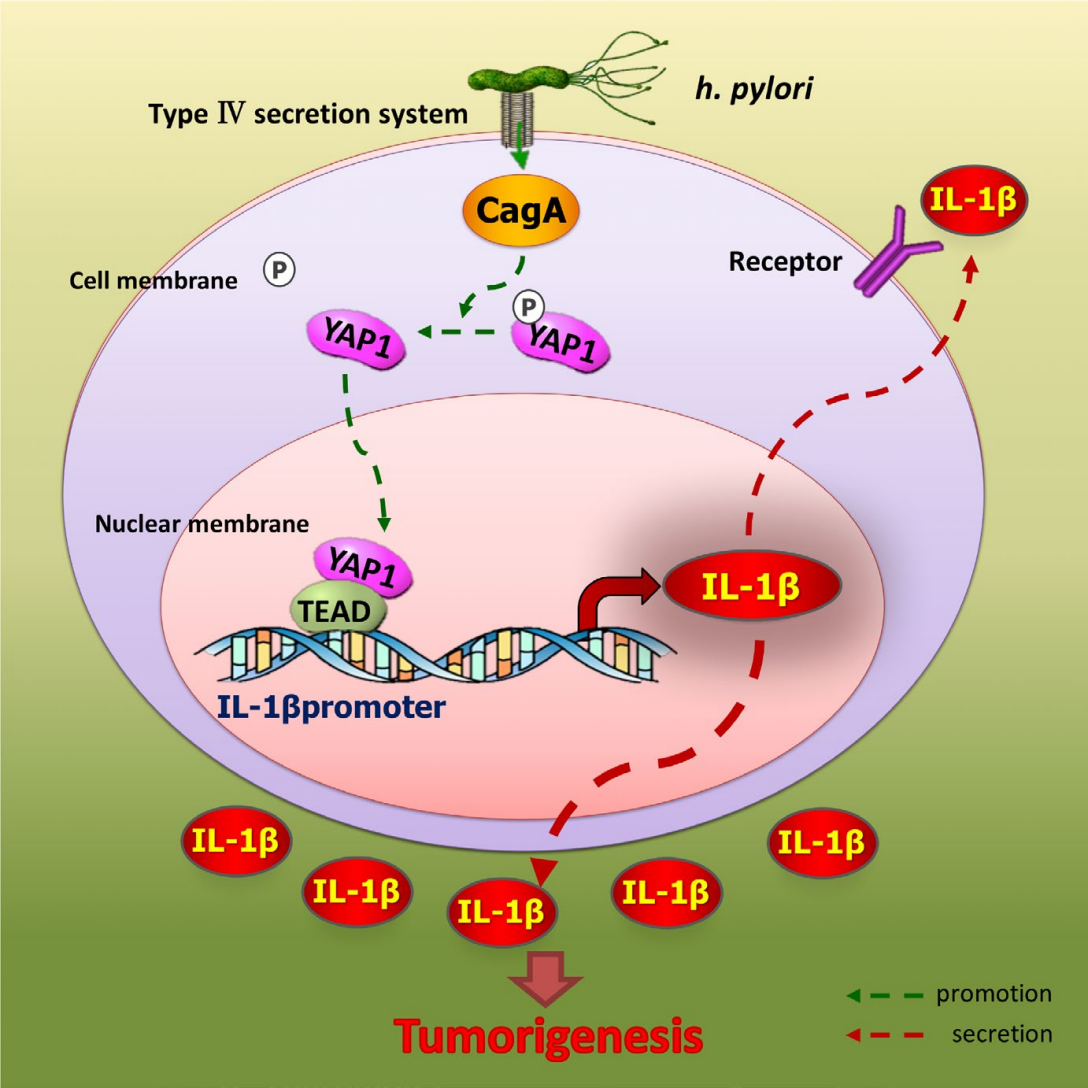
Overview of the *Hp*‐YAP1‐IL‐1β pathway in gastric carcinogenesis and development

To confirm the role of IL‐1β in YAP1‐regulated GC development, we introduced IL‐1β siRNA, and the knockdown efficiency was verified at the mRNA level (Figure S2E). IL‐1β si1 had the highest efficiency and was thus applied in the subsequent experiments. IL‐1β was knocked down in AGS and BGC‐823 cells after overexpressing YAP1S127A, and the colony formation assay was performed. IL‐1β knockdown compromised the positive effect of YAP1s127A on colony formation (Figure 6E). These results illustrated the important role of IL‐1β as a key downstream effector of YAP1 in GC cell proliferation. The *Hp*‐YAP1‐IL‐1β pathway may serve as a novel drug target in GC treatment (Figure 7A).

## 4 DISCUSSION

GC progression is a complex process that combines uncontrolled proliferation and unhealed inflammation, and the mechanism by which *Hp* infection induces gastric carcinogenesis is not fully elucidated. In this study, we demonstrate that *Hp* promotes tumor‐accelerating inflammation by enhancing YAP1 expression and nuclear translocation. YAP1 functions as a transcriptional co‐activator working together with TEAD. The two‐molecule complex binds the IL‐1β promoter and thereby increases IL‐1β expression. The abundant IL‐1β expression assists GC development by stimulating cancer cell proliferation. Taken together, *Hp* plays an important role in GC progression by regulating YAP1 and downstream IL‐1β.

The facts that higher incidence rates of GC occur in developing countries, such as China, and GC often develops from severe forms of gastritis indicates that hygienic standards, eating habits, and microbiota colonization are closely correlated with GC development.^1^, ^31^ Interactions between *Hp* and its hosts are rather complicated. *Hp* uses its bacterial type IV secretion system (T4SS) to inject the toxin CagA into its host's stomach epithelial cells, triggering a vast range of downstream signaling cascades and ultimately accelerating gastric carcinogenesis.^4^ Other toxins, such as VacA and CagL, as well as *Hp* components, such as LPS, also contribute to this malignant transformation. Recently, a group reported that *Hp* can promote GC by activating gastric stem cells.^32^ The gastric carcinogenesis initiated by *Hp* can be partially attributed to changes in signaling pathways, and the most studied pathways are toll‐like receptor‐4 (TLR‐4) and nuclear factor‐κβ (NF‐κB) signaling.^33^, ^34^Activation of TLR‐4 triggers a cascade of downstream signaling pathways, including phosphorylation of the mitogen‐activated protein kinase (MAPK) pathway, which contains the components extracellular signal‐regulated kinase (ERK), c‐Jun N‐terminal kinase (JNK), and p38. Phosphorylation of the MAKP pathway can further activate several key transcription factors and lead to uncontrolled growth in GC.^35^ Moreover, activation of the NF‐κB pathway also plays a critical role in maintaining cell proliferation and protecting cells from apoptosis in GC.^36^ Although not all mechanisms underlying the tumor‐promoting role of *Hp* have been well illustrated, *Hp* clearly and cleverly manipulates routine pathways in host cells as an external stimulus to ensure its survival and proliferation, as these pathways react sensitively to external signals. The Hippo cascade is a pathway that is sensitive to environmental signal perturbation.^5^ We observed an unreported phenomenon that the Hippo cascade key effector YAP1 exhibited increased nuclear translocation after gastric cells were infected with *Hp*, which aroused our interests to research new mechanisms underlying the role of YAP1 in *Hp*‐induced gastric carcinogenesis.

Despite the ability of inflammation to resist infection, the occasional unstoppable activation of inflammatory responses and activators creates a persistent inflammatory microenvironment that facilitates tumor‐promoting inflammation, which contributes to unresolved inflammation 37 and is recognized as a new cancer hallmark.^38^ As in some patients, *Hp *infection of the stomach can first cause chronic superficial gastritis, then chronic AG with intestinal metaplasia, then gastric dysplasia, and eventually GC,^18^, ^39^, ^40^ studying *Hp‐*induced gastric carcinogenesis is sufficient to illustrate how chronic inflammation and infection collaboratively trigger tumors. In our study, we attempted to elucidate the mechanism by which *Hp* infection triggers GC and how GC cells transform infection signals into constant inflammation, revealing that YAP1 serves as the link. YAP1 transcriptionally activates IL‐1β in GC cells, which is beyond the classic mechanism involving immune cell responses, and creates a complete inflammatory environment wherein the infection occurs.

Although the mechanisms by which inflammation promotes carcinogenesis remain unclear, some consensus has been reached among researchers regarding the inflammatory factors influencing carcinogenesis. Both IL‐1β and IL‐18 are proinflammatory cytokines that require inflammasomes to cleave unprocessed IL‐1β (pro IL‐1β) and unprocessed IL‐18 (pro IL‐18) into their biologically active forms.^41^ Once activated, with the help of caspase‐1, the mature IL‐1β and IL‐18 cause pyroptosis, a type of inflammatory cell death.^42^ In contrast to the constitutive expression of IL‐18, transcriptional activation of the pro IL‐1β gene is the foundation of IL‐1β activation, which is mainly mediated by NF‐κβ.^43^, ^44^ Activated IL‐1β provides positive feed‐forward stimulation for inflammatory cytokines, thereby amplifying inflammation, which can result in some diseases, including autoimmune diseases and cancer.^45^

Numerous studies have shown the positive role of IL‐1β in the progression of various cancers, including breast cancer, lung cancer, and hematological malignancies.^46^, ^47^, ^48^ Moreover, IL‐1β has been found to be the most relevant to GC development. Abnormally high IL1β expression is one of the most important factors accelerating GC progression. In our animal experiment, we observed reduced IL‐1β levels in the YAP1 knockdown group, which had an impaired tumorigenesis ability. In the *Hp* infection study, both YAP1 and IL‐1β expression showed an infection time‐dependent trend, which provided a clue into the novel mechanism. We also checked the expression of other inflammatory factors (IL‐6, IL8, IL‐10, and TNFα) in negative control and YAP1 knockdown groups in AGS and BGC. However, we did not find accordant results showing that they were YAP1 direct downstream effectors (Figure S2f).

TEADs are DNA‐binding proteins that can be activated by YAP1 and work as transcription factors.^49^ YAP1 has been demonstrated to promote tumorigenesis by activating TEAD in breast cancers, cholangiocarcinoma, and renal cell carcinoma.^50^, ^51^, ^52^ Moreover, TEADs were shown to be overexpressed in GC.^53^ In our study, disrupting the YAP1‐TEAD interaction interfered with the gastric tumor development process. The YAP1‐TEAD interaction can be disrupted by either adding the drug VP or transfecting mutant plasmids. In addition, an increasing number of studies have shown that VP has an anti‐proliferation effect on various cancers, including melanoma, prostate, liver, esophageal, and lung cancer.^54^, ^55^, ^56^, ^57^, ^58^ These findings indicate that YAP1 might become a new drug target in GC treatment, but further investigation is needed for clinical transformation.

In summary, our findings sketch an outline of the *Hp*‐YAP1‐IL‐1β pathway. This axis connects *Hp* infection to vast tissue inflammation and tumorigenesis and thereby plays an important role in GC initiation and development.

## CONFLICT OF INTEREST

The authors have no conflict of interest.

## AUTHOR CONTRIBUTIONS

JihuiJia, Jingya Yu and Yujiao Wu designed the study; Yujiao Wu, Jingya Yu, Xiuming Liang, Li Shen, Shuyan Li, and Lixin Zheng performed the study; Lin Ma, Tongyu Li, Han Yu and Hillary Chan analyzed the data; Chunyan Chen and JihuiJia supervised the study; and Yujiao Wu, Jingya Yu and JihuiJia wrote the manuscript.

## Data Availability

The data that support the findings of this study are available on request from the corresponding author. The data are not publicly available due to privacy or ethical restrictions.

## References

[1] Torre LA , Bray F , Siegel RL , Ferlay J , Lortet‐Tieulent J , Jemal A . Global cancer statistics, 2012. CA Cancer J Clin. 2015;65(2): 87‐108. 25651787 10.3322/caac.21262

[2] Uemura N , Okamoto S , Yamamoto S , et al. Helicobacter pylori infection and the development of gastric cancer. N Engl J Med. 2001;345(11):784‐789. 11556297 10.1056/NEJMoa001999

[3] Salama NR , Hartung ML , Muller A . Life in the human stomach: persistence strategies of the bacterial pathogen Helicobacter pylori. Nat Rev Microbiol. 2013;11(6):385‐399. 23652324 10.1038/nrmicro3016 PMC3733401

[4] Hatakeyama M . Linking epithelial polarity and carcinogenesis by multitasking Helicobacter pylori virulence factor CagA. Oncogene. 2008;27(55):7047‐7054. 19029944 10.1038/onc.2008.353

[5] Piccolo S , Dupont S , Cordenonsi M . The biology of YAP/TAZ: hippo signaling and beyond. Physiol Rev. 2014;94(4):1287‐1312. 25287865 10.1152/physrev.00005.2014

[6] Harvey KF , Zhang X , Thomas DM . The Hippo pathway and human cancer. Nat Rev Cancer. 2013;13(4):246‐257. 23467301 10.1038/nrc3458

[7] Taniguchi K , Wu LW , Grivennikov SI , et al. A gp130‐Src‐YAP module links inflammation to epithelial regeneration. Nature. 2015;519(7541):57‐62. 25731159 10.1038/nature14228 PMC4447318

[8] Mizuno T , Murakami H , Fujii M , et al. YAP induces malignant mesothelioma cell proliferation by upregulating transcription of cell cycle‐promoting genes. Oncogene. 2012;31(49):5117‐5122. 22286761 10.1038/onc.2012.5

[9] He C , Lv X , Hua G , et al. YAP forms autocrine loops with the ERBB pathway to regulate ovarian cancer initiation and progression. Oncogene. 2015;34(50):6040‐6054. 25798835 10.1038/onc.2015.52 PMC4580488

[10] Zhou D , Conrad C , Xia F , et al. Mst1 and Mst2 Maintain hepatocyte quiescence and suppress hepatocellular carcinoma development through inactivation of the Yap1 oncogene. Cancer Cell 2009;16(5):425–438. 19878874 10.1016/j.ccr.2009.09.026 PMC3023165

[11] Xu CM , Liu WW , Liu CJ , Wen C , Lu HF , Wan FS . Mst1 overexpression inhibited the growth of human non‐small cell lung cancer in vitro and in vivo. (1476–5500 (Electronic)). 10.1038/cgt.2013.40 23928732

[12] Vlug EJ , van de Ven R , Vermeulen JF , Bult P , van Diest PJ , Derksen P . Nuclear localization of the transcriptional coactivator YAP is associated with invasive lobular breast cancer. Cell Oncol. 2013;36(5):375–384. 10.1007/s13402-013-0143-7 PMC3777165 23949920

[13] Hu X , Xin Y , Xiao Y , Zhao J . Overexpression of YAP1 is correlated with progression, metastasis and poor prognosis in patients with gastric carcinoma. Pathol Oncol Res. 2014;20(4):805–811. 24643316 10.1007/s12253-014-9757-y

[14] Kang W , Tong JH , Chan AW , et al. Yes‐associated protein 1 exhibits oncogenic property in gastric cancer and its nuclear accumulation associates with poor prognosis. Clin Cancer Res. 2011;17(8):2130–2139. 21346147 10.1158/1078-0432.CCR-10-2467

[15] Li P , Sun D , Li X , et al. Elevated expression of Nodal and YAP1 is associated with poor prognosis of gastric adenocarcinoma. J Cancer Res Clin Oncol. 2016;142(8):1765–1773. 27325246 10.1007/s00432-016-2188-2 PMC4954832

[16] Sun D , Li X , He Y , et al. YAP1 enhances cell proliferation, migration, and invasion of gastric cancer in vitro and in vivo. Oncotarget. 2016;7(49):81062–81076. 27835600 10.18632/oncotarget.13188 PMC5348376

[17] El‐Omar EM , Carrington M , Chow WH , et al. The role of interleukin‐1 polymorphisms in the pathogenesis of gastric cancer. Nature. 2001;412(6842):99. 11808612 10.1038/35083631

[18] Fox JG , Wang TC . Inflammation, atrophy, and gastric cancer. J Clin Investig. 2007;117(1):60–69. 17200707 10.1172/JCI30111 PMC1716216

[19] Lanaya H , Natarajan A , Komposch K , et al. EGFR has a tumour‐promoting role in liver macrophages during hepatocellular carcinoma formation. Nat Cell Biol. 2014;16(10):972–977. 25173978 10.1038/ncb3031 PMC4183558

[20] Lasry A , Zinger A , Ben‐Neriah Y . Inflammatory networks underlying colorectal cancer. Nat Immunol. 2016;17(3):230–240. 26882261 10.1038/ni.3384

[21] Tu S , Bhagat G , Cui G , et al. Overexpression of interleukin‐1beta induces gastric inflammation and cancer and mobilizes myeloid‐derived suppressor cells in mice. Cancer Cell. 2008;14(5):408–419. 18977329 10.1016/j.ccr.2008.10.011 PMC2586894

[22] Hong JB , Zuo W , Wang AJ , Lu NH . Helicobacter pylori Infection synergistic with IL‐1beta gene polymorphisms potentially contributes to the carcinogenesis of gastric cancer. Int J Med Sci. 2016;13(4):298–303. 27076787 10.7150/ijms.14239 PMC4829543

[23] Huang FY , Chan AO , Rashid A , et al. Interleukin‐1beta increases the risk of gastric cancer through induction of aberrant DNA methylation in a mouse model. Oncology Lett. 2016;11(4):2919–2924. 10.3892/ol.2016.4296 PMC4812546 27073577

[24] Yin S , Lan C , Pei H , Zhu Z . Expression of interleukin 1beta in gastric cancer tissue and its effects on gastric cancer. OncoTargets Ther. 2016;9:31–35. 10.2147/OTT.S94277 PMC4694683 26730201

[25] Szasz AM , Lanczky A , Nagy A , et al. Cross‐validation of survival associated biomarkers in gastric cancer using transcriptomic data of 1,065 patients. Oncotarget. 2016;7(31):49322–49333. 27384994 10.18632/oncotarget.10337 PMC5226511

[26] Tang Z , Li C , Kang B , Gao G , Li C , Zhang Z . GEPIA: a web server for cancer and normal gene expression profiling and interactive analyses. Nucleic Acids Res. 2017;45(W1):W98–w102. 28407145 10.1093/nar/gkx247 PMC5570223

[27] Zhao B , Ye X , Yu J , et al. TEAD mediates YAP‐dependent gene induction and growth control. Genes Dev. 2008;22(14):1962–1971. 18579750 10.1101/gad.1664408 PMC2492741

[28] Liu‐Chittenden Y , Huang B , Shim JS , et al. Genetic and pharmacological disruption of the TEAD‐YAP complex suppresses the oncogenic activity of YAP. Genes Dev. 2012;26(12):1300–1305. 22677547 10.1101/gad.192856.112 PMC3387657

[29] Michels S , Schmidt‐Erfurth U . Photodynamic therapy with verteporfin: a new treatment in ophthalmology. Semin Ophthalmol. 2001;16(4):201–206. 15513441 10.1076/soph.16.4.201.10298

[30] Agostinis P , Berg K , Cengel KA , et al. Photodynamic therapy of cancer: an update. CA Cancer J Clin. 2011;61(4):250–281. 21617154 10.3322/caac.20114 PMC3209659

[31] Arnold M , Moore SP , Hassler S , Ellison‐Loschmann L , Forman D , Bray F . The burden of stomach cancer in indigenous populations: a systematic review and global assessment. Gut. 2014;63(1):64–71. 24153248 10.1136/gutjnl-2013-305033

[32] Sigal M , Logan CY , Kapalczynska M , et al. Stromal R‐spondin orchestrates gastric epithelial stem cells and gland homeostasis. Nature. 2017;548(7668):451–455. 28813421 10.1038/nature23642

[33] Slomiany BL , Slomiany A . Role of ghrelin‐induced phosphatidylinositol 3‐kinase activation in modulation of gastric mucosal inflammatory responses to Helicobacter pylori. Inflammopharmacology. 2014;22(3):169‐177 (1568–5608 (Electronic)). 24057979 10.1007/s10787-013-0190-8

[34] Slomiany BL , Slomiany A . Involvement of p38 MAPK‐dependent activator protein (AP‐1) activation in modulation of gastric mucosal inflammatory responses to Helicobacter pylori by ghrelin. Inflammopharmacology. 2013;21(1):67‐78 (1568–5608 (Electronic)). 22669511 10.1007/s10787-012-0141-9

[35] Mitsuno Y , Yoshida H , Maeda S , et alHelicobacter pylori induced transactivation of SRE and AP‐1 through the ERK signalling pathway in gastric cancer cells. Gut. 2001;49(1):18‐22. (0017–5749 (Print)). 11413105 10.1136/gut.49.1.18 PMC1728350

[36] Maeda S , Yoshida H , Ogura K , et al. H. pylori activates NF‐kappaB through a signaling pathway involving IkappaB kinases, NF‐kappaB‐inducing kinase, TRAF2, and TRAF6 in gastric cancer cells. Gastroenterology. 2000;119(1):97‐108 (0016–5085 (Print)). 10889159 10.1053/gast.2000.8540

[37] Nathan C , Ding A . Nonresolving inflammation. Cell. 2010;140(6):871–882. 20303877 10.1016/j.cell.2010.02.029

[38] Hanahan D , Weinberg RA . Hallmarks of cancer: the next generation. Cell. 2011;144(5):646–674. 21376230 10.1016/j.cell.2011.02.013

[39] Wang Y , Kato N , Hoshida Y , et al. Interleukin‐1beta gene polymorphisms associated with hepatocellular carcinoma in hepatitis C virus infection. Hepatology. 2003;37(1):65–71. 12500190 10.1053/jhep.2003.50017

[40] Houghton J , Wang TC . Helicobacter pylori and gastric cancer: a new paradigm for inflammation‐associated epithelial cancers. Gastroenterology. 2005;128(6):1567–1578. 15887152 10.1053/j.gastro.2005.03.037

[41] Elinav E , Nowarski R , Thaiss CA , Hu B , Jin C , Flavell RA . Inflammation‐induced cancer: crosstalk between tumours, immune cells and microorganisms. Nat Rev Cancer. 2013;13(11):759–771. 24154716 10.1038/nrc3611

[42] Guadagno J , Swan P , Shaikh R , Cregan SP . Microglia‐derived IL‐1beta triggers p53‐mediated cell cycle arrest and apoptosis in neural precursor cells. Cell Death Dis. 2015;6(6):e1779. (2041–4889 (Electronic)). 26043079 10.1038/cddis.2015.151 PMC4669832

[43] Latz E , Xiao TS , Stutz A . Activation and regulation of the inflammasomes. Nat Rev Immunol. 2013;13(6):397. (1474–1741 (Electronic)). 23702978 10.1038/nri3452 PMC3807999

[44] Vikhreva P , Petrova V , Gokbulut T , et al. TAp73 upregulates IL‐1β in cancer cells: Potential biomarker in lung and breast cancer? Biochem Biophys Res Commun. 2017;482(3):498‐505 (0006–291X (Print)). 28212736 10.1016/j.bbrc.2016.10.085 PMC5243147

[45] Strowig T , Henao‐Mejia J , Elinav E , Flavell R , Flavell R . Inflammasomes in health and disease. Nature. 2012;481(7381):278‐286. (1476–4687 (Electronic)). 22258606 10.1038/nature10759

[46] Jimenez‐Garduno AM , Mendoza‐Rodriguez MG , Urrutia‐Cabrera D , et al. IL‐1beta induced methylation of the estrogen receptor ERalpha gene correlates with EMT and chemoresistance in breast cancer cells. Biochem Biophys Res Commun. 2017;490(3):780‐785 (1090–2104 (Electronic)). 28645612 10.1016/j.bbrc.2017.06.117

[47] Arranz L , Arriero M , Villatoro A . Interleukin‐1beta as emerging therapeutic target in hematological malignancies and potentially in their complications. Blood Rev. 2017;31(5):306‐317 (1532–1681 (Electronic)). 28495184 10.1016/j.blre.2017.05.001

[48] Vikhreva P , Petrova V , Gokbulut T , et al. TAp73 upregulates IL‐1beta in cancer cells: Potential biomarker in lung and breast cancer? Biochem Biophys Res Commun. 2017;482(3):498‐505 (1090–2104 (Electronic)). 28212736 10.1016/j.bbrc.2016.10.085 PMC5243147

[49] Lin KC , Park HW , Guan KL . Regulation of the Hippo Pathway Transcription Factor TEAD. LID ‐ S0968–0004(17)30170–6 [pii] LID ‐ 10.1016/j.tibs.2017.09.003 [doi]. (0968–0004 (Print)). 10.1016/j.tibs.2017.09.003 PMC5735856 28964625

[50] Marti P , Stein C , Blumer T , et al. YAP promotes proliferation, chemoresistance, and angiogenesis in human cholangiocarcinoma through TEAD transcription factors. Hepatology. 2015;62(5):1497‐1510 (1527–3350 (Electronic)). 26173433 10.1002/hep.27992

[51] Wang C , Nie Z , Zhou Z , et al. The interplay between TEAD4 and KLF5 promotes breast cancer partially through inhibiting the transcription of p27Kip1. Oncotarget. 2015;6(19):17685‐17697. (1949–2553 (Electronic)). 25970772 10.18632/oncotarget.3779 PMC4627338

[52] Schutte U , Bisht S , Heukamp LC , et al. Hippo signaling mediates proliferation, invasiveness, and metastatic potential of clear cell renal cell carcinoma. Transl Oncol. 2014;7(2):309‐321 (1936–5233 (Print)). 24913676 10.1016/j.tranon.2014.02.005 PMC4101344

[53] Zhou GX , Li XY , Zhang Q , et al. Effects of the hippo signaling pathway in human gastric cancer. Asian Pac J Cancer Prev. 2013;14(9):5199‐5205. (2476–762X (Electronic)). 24175801 10.7314/apjcp.2013.14.9.5199

[54] Nguyen LT , Tretiakova MS , Silvis MR , et al. ERG Activates the YAP1 Transcriptional Program and Induces the Development of Age‐Related Prostate Tumors. Cancer Cell. 2015;27(6):797–808. 26058078 10.1016/j.ccell.2015.05.005 PMC4461839

[55] Yu FX , Luo J , Mo JS , et al. Mutant Gq/11 promote uveal melanoma tumorigenesis by activating YAP. Cancer Cell. 2014;25(6):822–830. 24882516 10.1016/j.ccr.2014.04.017 PMC4075337

[56] Weiler S , Pinna F , Wolf T , et al. Induction of Chromosome Instability by Activation of Yes‐Associated Protein and Forkhead Box M1 in Liver Cancer. Gastroenterology. 2017;152(8):2037–51.e22. 28249813 10.1053/j.gastro.2017.02.018

[57] Song S , Honjo S , Jin J , et al. The Hippo coactivator YAP1 mediates EGFR overexpression and confers chemoresistance in esophageal cancer. Clin Cancer Res. 2015;21(11):2580–2590. 25739674 10.1158/1078-0432.CCR-14-2191 PMC4452384

[58] Dai Y , Liu S , Zhang WQ , et al. YAP1 regulates ABCG2 and cancer cell side population in human lung cancer cells. Oncotarget. 2017;8(3):4096–4109. 27911857 10.18632/oncotarget.13686 PMC5354815

