# Supplementary material for: Helicobacter pylori‐induced YAP1 nuclear translocation promotes gastric carcinogenesis by enhancing IL‐1β expression
Source: Cancer Med. 2019 May 30;8(8):3965–80. doi: 10.1002/cam4.2318 (PMC6639191; doi:10.1002/cam4.2318)
Supplement: Supplementary file 1 [file CAM4-8-3965-s001.docx]

Supplementary data

S1(a) immunohistochemistry (IHC) results showed that YAP1 is highly expressed in *helicobacter pylori* (*hp*) positive atrophic gastritis (AG) patients.

(b) Hematoxylin and eosin (HE) staining showed that more lymphocytes (black arrows) were observed in negative control (nc) group than shYAP1 group in xenograft mice.

S2(a) Western blot showing YAP1 expression levels in GES-1, AGS and BGC cells.

(b) The YAP1 siRNA knockdown efficiency was verified in BGC-823 cells.

(c) Western blot confirming the efficiencies of the YAP1S127A and YAP1up plasmids in BGC-823 cells.

(d) Immunofluorescence staining of BGC-823 cells transfected with control and YAP1S127A plasmids. The YAP1S127A plasmid can provide the serine 127 mutant form YAPS127, which can be transported into the nucleus.

(e) The IL-1β siRNA knockdown efficiency was verified by qPCR (data are presented as the mean ± SD, ***=p<0.001).

(f) IL-6, IL-8, IL-10 and TNFα mRNA expression in negative control (nc) and siYAP1 group (data are presented as the mean ± SD, ***=p<0.001).
